# The molecular interplay between the gut microbiome and circadian rhythms: an integrated review

**DOI:** 10.3389/fmicb.2025.1712516

**Published:** 2025-12-05

**Authors:** Boyang Zheng, Liwei Wang, Shilin Sun, Xingxing Yuan, Qun Liang

**Affiliations:** 1 Graduate School, Heilongjiang University of Chinese Medicine, Harbin, China; 2 Department of Medicine, Heilongjiang Academy of Traditional Chinese Medicine, Harbin, China; 3 Department of Critical Care Medicine, First Affiliated Hospital of Heilongjiang University of Chinese Medicine, Harbin, China

**Keywords:** gut microbiome, circadian rhythms, microbial metabolites, chronodisruption, time-restricted eating, lithocholic acid, tryptophan derivatives, probiotic

## Abstract

This integrated review synthesizes current evidence on the molecular interplay between the gut microbiome and circadian rhythms, emphasizing a sophisticated bidirectional communication system crucial for maintaining metabolic, immune, and neurological homeostasis. The host circadian clock orchestrates microbial composition and function through rhythmic changes in feeding-fasting cycles, hormone secretion, immune responses, and bile acid metabolism. In return, microbial metabolites, including short-chain fatty acids such as butyrate, secondary bile acids like lithocholic acid, and tryptophan derivatives, act as timing cues that influence core clock gene expression via epigenetic mechanisms, receptor-mediated signaling (GPR41/43, FXR), and neuroendocrine pathways. Disruption of this finely tuned dialogue, known as chronodisruption, often driven by modern lifestyles, predisposes individuals to a range of pathologies, including metabolic syndrome, inflammatory bowel disease (IBD), neurodegenerative disorders, and cancer. The review also evaluates promising chronotherapeutic interventions such as time-restricted eating (TRE), targeted probiotic use, and chronopharmacology, which aim to resynchronize host-microbe rhythms and restore physiological balance. Elucidating these mechanisms provides a foundational framework for developing personalized health strategies that target the gut-clock axis.

## 1 Introduction

The intricate symphony of life is conducted by two fundamental orchestrators: the circadian clock, an evolutionarily conserved timekeeping system that synchronizes physiological processes with the Earth's 24-hour light-dark cycle, and the gut microbiome, a vast and dynamic ecosystem of trillions of microorganisms residing within the gastrointestinal tract. For decades, these systems were studied in isolation by chronobiologists and microbiologists, respectively. However, a paradigm shift has unfolded over the past decade, fundamentally reshaping our understanding of human physiology. This shift was catalyzed by a series of pioneering studies in the mid-2010s that provided the first robust evidence of a profound, bidirectional molecular dialogue between the host's circadian rhythms and the commensal gut microbiota (Thaiss et al., 2014; Zarrinpar et al., 2014; Leone et al., 2015). We now understand that this crosstalk is not merely associative but is mechanistic, underpinning core aspects of metabolism, immune function, neuronal activity, and overall health. Consequently, the disruption of this delicate equilibrium a state termed chronodisruption is increasingly implicated in the pathogenesis of a wide array of disorders, including metabolic syndrome, inflammatory bowel disease (IBD), neurodegenerative diseases, cancer, and psychiatric conditions (Yang et al., 2023a; Moreno-Cortes et al., 2024; Swanton et al., 2024; Zhou et al., 2024).

Epidemiological trends highlight the global burden of diseases linked to chronodisruption and gut dysbiosis. Countries with highly industrialized and urbanized populations, such as the United States, China, and Mexico, often report among the highest incidence rates of metabolic syndrome and type 2 diabetes mellitus (T2DM). Similarly, nations like Canada and those in Northern Europe show a high prevalence of IBD. The rising incidence of neurodegenerative disorders is a significant concern in aging populations across Western Europe, North America, and Japan. Concurrently, modern lifestyles in these regions are characterized by significant circadian misalignment. For instance, population studies indicate that adults in countries like Japan, South Korea, and Saudi Arabia report some of the shortest average sleep durations and a high prevalence of habitual short sleep, a proxy for staying up late. In China and the United States, a substantial portion of the population, particularly among younger adults and shift workers, regularly experiences short sleep cycles and social jet lag, spending an average of 2–3 nights per week with insufficient sleep due to late-night activities. This correlation between regions with high disease burden and populations experiencing chronic sleep curtailment underscores the potential public health impact of the gut-clock axis dysregulation (Heddes et al., 2022; Tofani et al., 2025).

The central thesis unifying the research presented herein is that the gut microbiome and the host circadian system do not operate in parallel but form an integrated, super organismal network. They engage in continuous, reciprocal communication through a complex web of molecular signals. This dialogue is essential for maintaining metabolic homeostasis, immune competence, and neurological function. The core premise rests on a two-way street of influence. First, the host's circadian clocks, both the central pacemaker in the suprachiasmatic nucleus (SCN) and peripheral oscillators in organs like the liver and gut, exert top-down control over the microbial community. This regulation is mediated through cyclical changes in host physiology, including feeding-fasting cycles, body temperature fluctuations, hormone secretion (glucocorticoids and melatonin), immune responses, and the synthesis and secretion of bile acids and antimicrobial peptides(Fowler et al., 2022; Wang et al., 2022; Litwin and Koronowski, 2025; Mortimer et al., 2025; Sciarra et al., 2025; Zhu et al., 2025). These rhythmic host outputs create a dynamic environment in the gut lumen, selectively favoring microbial species that can best utilize available resources at specific times of day, thereby driving daily oscillations in microbial composition and function.

Concurrently, and with equal importance, the microbiome and its vast metabolome act from the bottom up, generating potent timing cues that influence and entrain host circadian pathways. Key microbial metabolites, including short-chain fatty acids (SCFAs) like butyrate from dietary fiber fermentation, bacterially modified secondary bile acids such as lithocholic acid (LCA), and tryptophan derivatives like indole serve as epigenetic modifiers, receptor ligands, and neuroendocrine signals. They have been shown to influence the expression and function of core clock genes and their output pathways in host tissues via mechanisms such as histone deacetylase (HDAC) inhibition, activation of G-protein-coupled receptors (GPR41/43, TGR5) and nuclear receptors (FXR, AhR), and modulation of neuroendocrine circuits (Kubo, 2020; Pi et al., 2020; Gutierrez Lopez et al., 2021; Khezri et al., 2023; Li et al., 2024; Sharma et al., 2025). The discovery that a microbiome-derived bile acid can directly stabilize the core clock protein CRY2 exemplifies the profound specificity of this molecular crosstalk (Kubo, 2020). Modern lifestyle factors such as shift work, exposure to artificial light at night (ALAN), social jet lag, and erratic eating patterns- act as potent disruptors of this finely tuned bidirectional axis, creating a state of misalignment between our internal clocks and the external environment that serves as a key contributor to the pathophysiology of numerous chronic diseases (Papavasileiou et al., 2023; Xu et al., 2025a,b).

The rationale for meticulously dissecting this molecular interplay is both profound and multifold. Firstly, the systems involved are master regulators of physiology. The circadian clock is estimated to regulate the transcription of up to 80% of protein-coding genes in mammals, including those governing metabolism, cell cycle, DNA repair, and inflammation (Bolshette et al., 2023; Sato and Sato, 2023). Similarly, the gut microbiome is often described as a virtual endocrine organ, producing a plethora of metabolites that influence host physiology far beyond the confines of the gut, reaching the liver, brain, and other distal organs (Su et al., 2025; Yuan et al., 2025a). The convergence of these two powerful systems suggests a critical node for physiological regulation. Secondly, epidemiological and clinical observations consistently demonstrate that circadian disruptors, such as jet lag and rotating shift work, are strongly associated with microbial dysbiosis an imbalance in the microbial community and a significantly increased risk for conditions like obesity, T2DM, and cardiovascular disease (Ansu Baidoo and Knutson, 2023; Duez and Staels, 2025; Nuszkiewicz et al., 2025). Conversely, interventions that alter the microbiome, such as broad-spectrum antibiotic use or drastic dietary shifts, have been shown to impair circadian rhythmicity in the host, underscoring the reciprocity of this relationship (Patangia et al., 2022; Lathakumari et al., 2024).

Furthermore, controlled animal models provide compelling causal evidence that moves beyond human correlation. Studies using genetically circadian-disrupted mice (*Clock* mutant or *Bmal1* knockout models) consistently demonstrate altered microbial communities and exacerbated disease phenotypes upon metabolic or inflammatory challenge (Mistry et al., 2020; Frazier et al., 2023; Jochum et al., 2023). Germ-free mice, which lack a microbiome entirely, show profoundly abnormal circadian rhythms in gene expression and metabolic pathways; these abnormalities can be partially restored by microbial colonization or even by the administration of specific microbial metabolites (Frazier et al., 2020; Pi et al., 2020). However, the translational interpretation of these findings requires caution. Critical limitations, such as the fundamental differences between nocturnal rodents and diurnal humans, divergent gut physiology, and distinct bile acid metabolism, must be acknowledged. These differences mean that mechanistic conclusions from animal studies, while invaluable, should be contextualized as potential pathways rather than established facts for human applicability.

Elucidating the molecular mechanisms connecting the microbiome and circadian rhythms is of paramount significance for several pressing reasons: Modern society is characterized by widespread chronodisruption. Understanding how this misalignment propagates through the microbiome to affect health could inform public health strategies and occupational guidelines to mitigate the risks associated with shift work, excessive screen time, and irregular lifestyles. This field opens the door to innovative “chrono-microbiome” therapies. These could include chrononutrition (time-restricted eating, TRE), timing the administration of drugs, probiotics, or prebiotics to coincide with peaks in host and microbial metabolic cycles (chronopharmacology and chronobiotics), and leveraging the microbiome to reinforce robust circadian rhythms (Hu et al., 2020; Ratiner et al., 2022; Weger et al., 2023; Hein et al., 2025; Smith et al., 2025). Many diseases with incompletely understood etiologies, such as IBD, neurodegenerative disorders like Alzheimer's and Parkinson's disease, and certain cancers, show concurrent disruptions in both circadian rhythms and the gut microbiome (Yang and Zhang, 2020; Khezri et al., 2023; Yang et al., 2023b; Liu et al., 2024). Deciphering their molecular interplay provides a unifying framework to understand disease pathogenesis and identify novel therapeutic targets.

This comprehensive review integrates recent literature to achieve four key objectives: first, to delineate the mechanisms by which the host circadian system regulates the gut microbiome through rhythmic control of feeding-fasting cycles, intestinal motility, immune function, and secretion of bile acids and antimicrobial peptides. Second, to explore in detail the molecular pathways through which microbial metabolites influence host circadian biology, focusing on SCFAs, bacterially modified bile acids, and neuroactive metabolites. Third, to examine the pathological consequences of axis dysregulation across a spectrum of diseases, including metabolic diseases, gastrointestinal disorders, neurodegenerative conditions, and cancer. And finally, to critically discuss emerging therapeutic strategies including chrononutrition, prebiotic/probiotic supplementation, fecal microbiota transplantation (FMT), and chronopharmacology, while rigorously evaluating their potential and their significant translational hurdles based on the current clinical evidence (Hu et al., 2020; Li et al., 2020; Frazier et al., 2023; Chen et al., 2024; He et al., 2024; Godos et al., 2025; Priya et al., 2025).

This integrated review aims to synthesize the current understanding of the molecular mechanisms that facilitate this complex relationship. To ensure a comprehensive, transparent, and reproducible synthesis of the literature, we conducted a systematic search of major academic databases, including PubMed, Scopus, and Web of Science, between 2023 and 2025. Our search strategy employed a combination of keywords and Medical Subject Headings (MeSH) terms related to the core concepts: (“gut microbiome” OR “microbiota” OR “microbiome”) AND (“circadian rhythm” OR “biological clock” OR “clock gene” OR “BMAL1” OR “CLOCK” OR “PER” OR “CRY”) AND (“molecular mechanism” OR “metabolite” OR “short-chain fatty acid” OR “SCFA” OR “bile acid” OR “tryptophan” OR “signaling”). The initial search results were screened by title and abstract, followed by a full-text review of relevant articles. Inclusion criteria prioritized peer-reviewed original research articles, systematic reviews, and meta-analyses that provided direct mechanistic insights into the gut-microbiome-circadian axis. This methodology was designed to minimize selection bias and provide a balanced overview of the field's current landscape.

**Figure 1:**
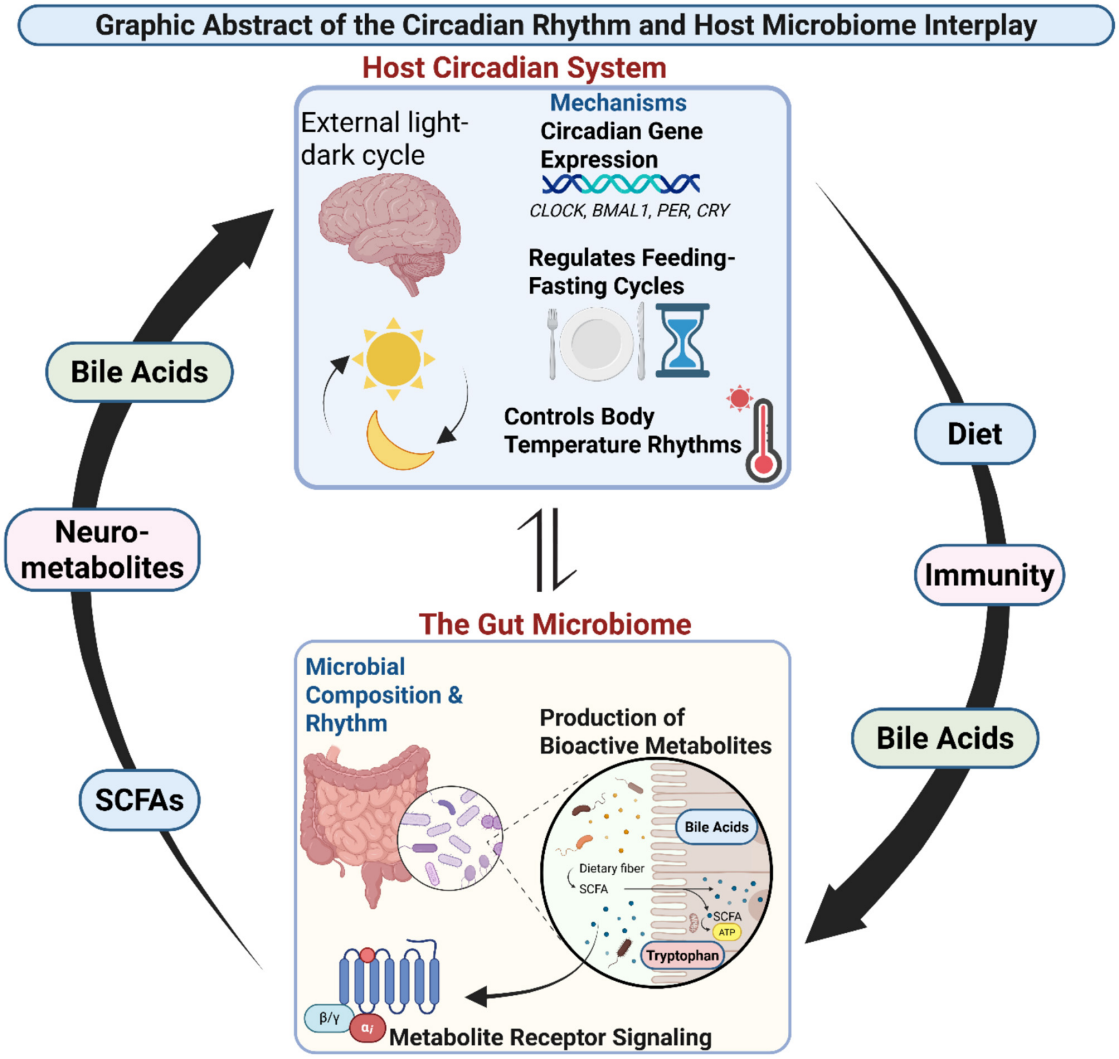
Graphic abstract of the circadian rhythm and host-microbiome interplay. The host circadian system, regulated by the external light-dark cycle, drives circadian gene expression (CLOCK, BMAL1, PER, CRY), which in turn regulates feeding-fasting cycles and body temperature rhythms. These circadian outputs shape gut microbial composition and rhythms, influencing the production of bioactive metabolites such as bile acids, SCFAs, and tryptophan derivatives. Microbial metabolites act through receptor signaling to impact host physiology, feeding back into circadian regulation. This bidirectional interaction integrates with host immunity, diet, and metabolic pathways, highlighting the dynamic crosstalk between host circadian rhythms and the gut microbiome.

The molecular crosstalk between the gut microbiome and circadian rhythms represents a fundamental biological principle that integrates environmental cues, such as light and food, with host physiology. By synthesizing the current evidence, this review will provide a detailed overview of this dynamic interface, highlighting its critical role in health and disease and outlining the promising, yet challenging, frontier of chrono-microbiome-targeted interventions (Figure 1).

## 2 The molecular interplay between the gut microbiome and circadian rhythms

The dialogue between the host circadian system and the gut microbiome is a continuous, bidirectional exchange of signals. This Section dissects this interplay into its constituent directions-host-to-microbiome and microbiome-to-host synthesizing evidence from both animal and human studies. A critical analysis of the translational challenges inherent in this evidence is integrated throughout, moving beyond mere description to a balanced evaluation of the mechanistic claims. The subsequent parts will examine the pathological consequences of dysregulation and the therapeutic strategies emerging from this knowledge.

### 2.1 Host-to-microbiome: how the circadian clock shapes the gut ecosystem

The host circadian system exerts top-down control over the gut microbiome through several key rhythmic physiological outputs, creating a dynamic habitat that selects for a specific microbial community structure and function. The most potent entraining signal for peripheral clocks, including those in the gastrointestinal tract, is the timing of food intake. The host's circadian-driven feeding behavior creates rhythmic fluctuations in nutrient availability within the gut lumen. This, in turn, imposes a selective pressure that favors microbial taxa capable of utilizing these resources at specific times of the day. Robust evidence from mouse models shows that up to 20% of bacterial taxa oscillate in abundance over a 24-hour period, synchronously with host feeding rhythms (Gutierrez Lopez et al., 2021). For instance, the relative abundances of *Firmicutes* and *Bacteroidetes* can shift between active and rest phases. Disruption of these cycles, through mistimed feeding or ad libitum access to high-fat diets, rapidly dampens these microbial oscillations and promotes dysbiosis (Yuan et al., 2025b; Zhu et al., 2025). It is crucial to note, however, that while these rhythms are clear in controlled animal studies, demonstrating equally robust and consistent rhythms in free-living humans, with their diverse diets and lifestyles, remains a methodological challenge.

#### 2.1.1 Rhythmic host immune and endocrine secretions

The circadian clock tightly regulates the secretion of antimicrobial peptides (AMPs), immunoglobulins (notably IgA), and key hormones such as glucocorticoids and melatonin (Fowler et al., 2022; Sciarra et al., 2025). These factors create a rhythmic immunological and endocrine landscape within the gut that directly influences microbial growth, gene expression, and community structure. For example, rhythmic IgA coating helps maintain microbial homeostasis and protects against pathobionts. Similarly, glucocorticoid rhythms can modulate microbial composition and the host's inflammatory response to the microbiota. The hormone melatonin, whose production is exquisitely light-sensitive, also exhibits circadian rhythms that can modulate gut motility and the microbial community; its disruption has been linked to dysbiosis in both animal and human studies (Khezri et al., 2023; Han et al., 2024). The challenge lies in delineating the specific contribution of each of these rhythmic signals from the overarching effect of feeding cycles.

#### 2.1.2 Circadian regulation of gut motility and permeability

Intestinal permeability and peristalsis are under direct circadian control. Rhythmic changes in gut motility alter the transit time of luminal contents, thereby affecting the niches available for different microbial species to colonize and proliferate. Disruption of the core clock, as seen in sleep deprivation or jet lag models, frequently leads to increased gut permeability (“leaky gut”), a pro-inflammatory state, and dysbiosis (Jochum et al., 2023; Yang et al., 2023a). This creates a feed-forward loop, where circadian disruption impairs barrier function, leading to inflammation that further perturbs both the clock and the microbiome.

#### 2.1.3 Hepatic control of bile acid metabolism

The liver clock regulates the rhythmic synthesis and secretion of bile acids (BAs), which are potent antimicrobial agents. The daily pulse of BAs into the duodenum and ileum shapes the microbial community in the upper GI tract, selecting for BA-resistant organisms. Circadian disruption, such as in shift work models, alters both the composition and the rhythmicity of the bile acid pool, which in turn drives a remodeling of the microbiome (Gumz et al., 2023; Zhu et al., 2025). A critical translational hurdle here is the significant difference between the bile acid pools of mice and humans; the specific bacterial transformations and the resulting secondary bile acids can vary, raising questions about the direct applicability of mechanistic findings involving, for example, specific microbially modified bile acids (Pi et al., 2020; Yang and Zhang, 2020).

### 2.2 Microbiome-to-host: microbial metabolites as circadian zeitgebers

In return, the gut microbiome influences host circadian rhythms and physiology primarily through its diverse repertoire of metabolites, which act as signaling molecules to entrain peripheral clocks and modulate systemic functions. The evidence for this is compelling but requires careful interpretation, especially when relying on pre-publication data.

#### 2.2.1 SCFAs

Acetate, propionate, and butyrate, produced by bacterial fermentation of dietary fiber, are key mediators. Butyrate, in particular, has been shown to entrain peripheral clocks by inhibiting histone deacetylases (HDACs), leading to chromatin remodeling and enhanced expression of core clock genes such as *Per2* (Li et al., 2024; Sharma et al., 2025). Furthermore, SCFAs activate G-protein-coupled receptors (GPR41/43) on enteroendocrine cells, stimulating the time-dependent release of peptides like PYY and GLP-1, which influence feeding behavior, insulin sensitivity, and energy homeostasis (Gutierrez Lopez et al., 2021). There is also emerging evidence that SCFAs can influence the central nervous system either by crossing the blood-brain barrier or by signaling via the vagus nerve, thereby potentially modulating central rhythms and sleep (Alachkar et al., 2022). However, the physiological relevance of the concentrations required for these effects, particularly HDAC inhibition, in vivo remains an active area of investigation.

#### 2.2.2 Bile acids (BAs)

Microbiota-modified secondary bile acids serve as potent signaling molecules that activate the nuclear receptor FXR and the membrane receptor TGR5. This activation regulates circadian-controlled glucose, lipid, and energy metabolism (Gasmi et al., 2025; Zhu et al., 2025). Evidence has revealed a novel and direct mechanism whereby the secondary bile acid LCA lengthens the circadian period by directly stabilizing the core clock protein CRY2 (Pi et al., 2020). While this finding points to a groundbreaking direct molecular link, it is critical to highlight that this study is currently a preprint and has not yet undergone peer review. Therefore, its conclusions must be treated as preliminary and require independent validation before being accepted as an established mechanism.

#### 2.2.3 Tryptophan derivatives

Tryptophan metabolism by gut bacteria produces a range of bioactive compounds, including serotonin and indole derivatives. These metabolites regulate gastrointestinal functions like motility through circadian processes and influence the gut-brain axis by activating the aryl hydrocarbon receptor (AhR) (Khezri et al., 2023; Siebieszuk et al., 2023). The AhR can interact with the CLOCK-BMAL1 complex, providing a potential pathway for microbial metabolites to directly influence circadian transcription and immune regulation. The balance between serotonin synthesis (which can promote wakefulness) and melatonin synthesis (which promotes sleep) in the gut is also a fascinating, though complex, aspect of this regulation.

#### 2.2.4 Other metabolites

Additional microbial products, including polyamines, vitamins, and even structural components like lipopolysaccharide (LPS), exhibit diurnal rhythms that can influence host circadian physiology, immune function, and metabolic status, further illustrating the breadth of this microbial influence.

### 2.3 Consequences of dysregulation: the path to disease

When the harmonious dialogue between the microbiome and the circadian clock is disrupted, it creates a self-perpetuating state of dysbiosis and chronodisruption that fuels disease pathogenesis across multiple organ systems.

#### 2.3.1 Metabolic disease

The liver, a key metabolic organ, is tightly regulated by both the circadian clock and microbial signals. Disruption of this axis is a primary driver of obesity and T2DM. High-fat diet (HFD) feeding in mice causes rapid dysbiosis and flattens circadian rhythms in clock gene expression within metabolic tissues like the liver and adipose tissue (Yuan et al., 2025b). This leads to impaired glucose tolerance, insulin resistance, and hepatic steatosis. HFD also alters bile acid composition, disrupting FXR signaling. Combined with a loss of rhythmic SCFA production, this impairs the circadian coordination of critical metabolic pathways like gluconeogenesis and lipid oxidation (Frazier et al., 2023). Conversely, time-restricted feeding, which enforces a daily fasting period, can restore microbial rhythms, improve SCFA production, and protect against metabolic disease, even under a HFD, primarily by reinstating a robust circadian metabolic program (Hu et al., 2020). The striking efficacy of time-restricted feeding in rodent models, however, is often contrasted with the more modest and variable outcomes in human trials, where adherence and individual variability play a major role.

#### 2.3.2 Gastrointestinal disorders

The intestinal epithelium has its own circadian clock that regulates proliferation, barrier function, and immune responses. Mice with an intestinal epithelial-specific knockout of *Bmal1* develop more severe colitis in response to dextran sulfate sodium (DSS) (Jochum et al., 2023). This pathology is associated with a loss of beneficial SCFA-producing bacteria, reduced SCFA levels, and heightened activation of pro-inflammatory STAT3 signaling. This demonstrates a direct causal link where circadian dysfunction within the gut itself predisposes to inflammation via microbiome-mediated mechanisms. While this provides a powerful mechanistic model for IBD, the direct translation to human IBD, a heterogenous disease, requires further validation.

#### 2.3.3 Neurodegenerative and neuropsychiatric disorders

The microbiota-gut-brain axis is heavily influenced by circadian rhythms. Sleep deprivation, a potent circadian disruptor, promotes the accumulation of amyloid-beta (Aβ) and tau pathology in models of Alzheimer's Disease (AD) through mechanisms that include impaired glymphatic clearance, glial cell activation, and microbiome-driven neuroinflammation (Gumz et al., 2023). TRE has shown promise in AD models by reducing Aβ deposition and modulating neuroinflammation (Moreno-Cortes et al., 2024). In Parkinson's Disease (PD), circadian disruption is both a symptom and a potential contributor to pathogenesis, with clock gene dysregulation potentially exacerbating mitochondrial dysfunction and neuroinflammation, potentially influenced by the microbiome via the gut-brain axis (Khezri et al., 2023; Chen et al., 2024). Similarly, circadian rhythm disruptions and gut dysbiosis are common features of major depressive disorder and anxiety, involving pathways like altered serotonin metabolism and HPA axis dysregulation (Mu et al., 2025; Priya et al., 2025). It is important to note that much of the evidence linking specific microbes to brain pathology is derived from animal models, and establishing direct causality in humans remains a significant challenge.

#### 2.3.4 Cancer

Circadian disruption is a recognized risk factor for cancer, and the microbiome is emerging as a key player. For instance, the oncogene PRMT6 can form a complex to repress the core clock gene *PER3*, thereby disrupting circadian rhythms and promoting cancer proliferation (Liu et al., 2024). More directly, a recent study demonstrated that circadian dysfunction can accelerate colorectal cancer (CRC) metastasis by promoting the accumulation of myeloid-derived suppressor cells (MDSCs) in the lungs. Gut microbiota-derived taurocholic acid (TCA) was identified as a key metabolite driving this process by enhancing MDSC glycolysis and immune suppression (Yang and Zhang, 2020). This illustrates how a dysregulated circadian-microbiome axis can create a permissive systemic environment for cancer progression. The translation of these findings will require determining if similar pathways are active in human cancers and if they can be therapeutically targeted.

### 2.4 Therapeutic strategies and future directions: a critical appraisal

Understanding this molecular interplay opens up novel therapeutic avenues focused on resynchronizing the host-microbiome dialogue. However, a balanced perspective that weighs promising mechanistic evidence against current clinical realities is essential.

#### 2.4.1 TRE

TRE remains the most promising and accessible intervention. By consolidating food intake to a consistent 8–12-h window, TRE reinforces circadian rhythms in the gut and liver, promotes a healthier microbiome structure (often enriching for SCFA-producers), and improves metabolic outcomes in both animal and human studies (Hu et al., 2020; Li et al., 2020). Its power lies in simultaneously engaging both the host clock and the microbial partner. However, long-term adherence in free-living populations is a major hurdle, and the “one-size-fits-all” approach may not be optimal, suggesting a future need for personalized feeding windows based on chronotype and microbiome.

#### 2.4.2 Prebiotics, probiotics, and fecal microbiota transplantation

Targeted interventions with specific fibers (prebiotics) or beneficial bacteria (probiotics) aim to support circadian health. For example, certain *Lactobacillus* strains have been shown to improve sleep and reduce anxiety in rodent models (Alachkar et al., 2022; Du et al., 2025). However, human probiotic trials have notoriously mixed results, often failing to replicate the robust effects seen in animals due to challenges in bacterial engraftment and the context-dependence of probiotic function within a complex, pre-existing microbial community. The concept of “chronobiotics” probiotics engineered to produce circadian-modulating metabolites in a time-controlled manner is intriguing but firmly in the experimental stage (Zhang et al., 2023). Similarly, while FMT from healthy donors can transfer microbial rhythms and confer metabolic benefits in model systems, demonstrating its efficacy for circadian-related disorders in humans is preliminary, and significant safety and regulatory hurdles remain (He et al., 2024; Zhang et al., 2025).

#### 2.4.3 Chronopharmacology and targeted drug development

**Figure 2:**
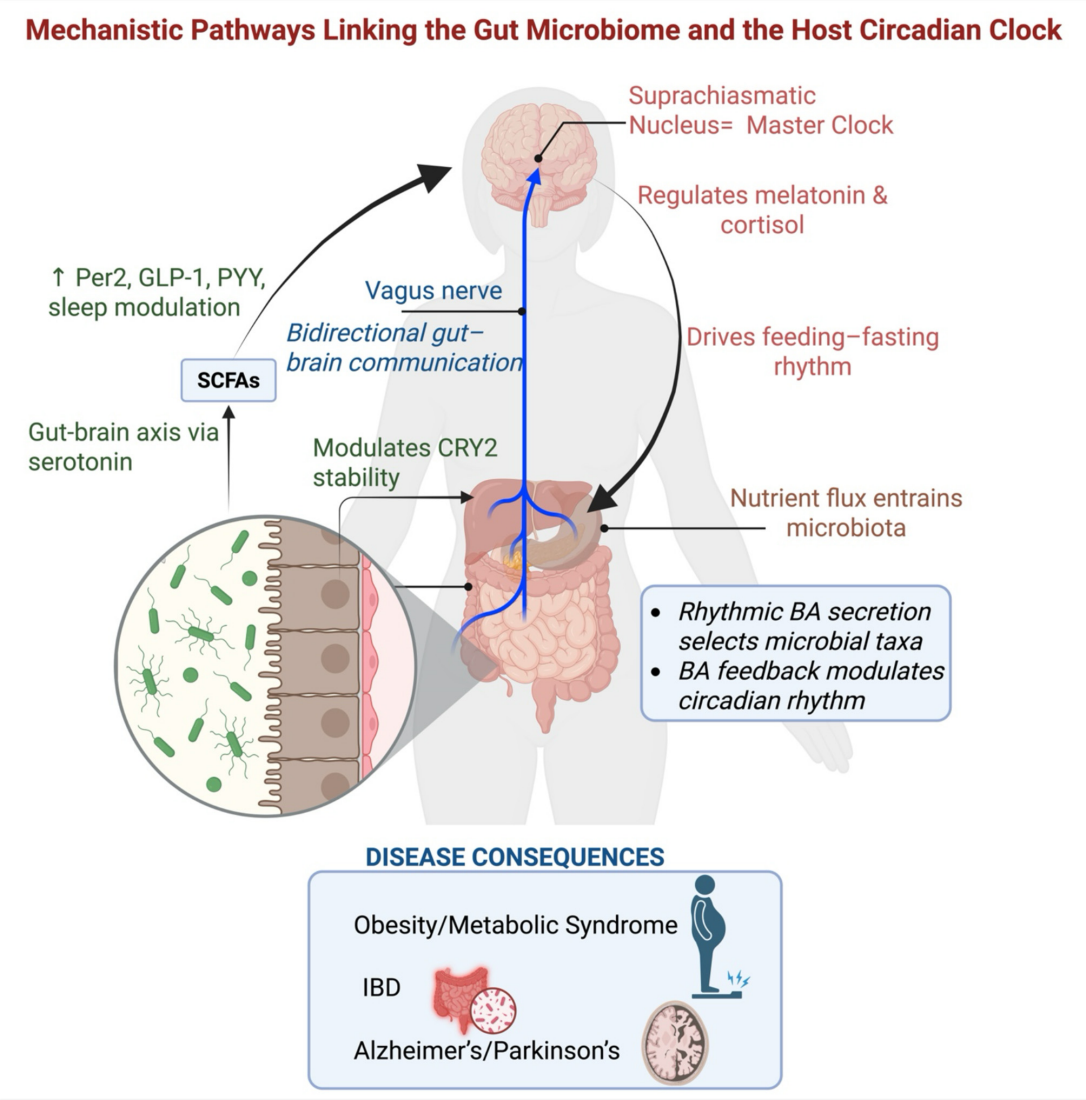
Mechanistic pathways linking the gut microbiome and the host circadian clock. This figure illustrates the bidirectional molecular and physiological communication between the host circadian system and the gut microbiota. The suprachiasmatic nucleus (SCN), the master circadian clock in the brain, regulates endocrine rhythms such as melatonin and cortisol and entrains feeding-fasting cycles, which in turn drive rhythmic nutrient availability in the gut and influence microbial community structure. Rhythmic bile acid (BA) secretion, under hepatic clock control, further selects for specific microbial taxa, while microbial metabolism of bile acids generates feedback signals that modulate host circadian gene expression, including CRY2 stability. Gut-derived SCFAs and tryptophan metabolites such as serotonin influence host clocks via epigenetic and neuroendocrine mechanisms. Additionally, the vagus nerve mediates bidirectional gut–brain communication, relaying microbial signals to central clock systems and influencing systemic physiology. Dysregulation of this host-microbe circadian axis contributes to pathogenesis in a range of disorders, including obesity and metabolic syndrome, IBD, and neurodegenerative diseases such as Alzheimer's and Parkinson's disease.

The principles of chronopharmacology timing drug administration to coincide with circadian rhythms in drug metabolism, target availability, and toxicity are well established. Applying this to consider the patient's circadian phase and the rhythmicity of their microbiome could further maximize therapeutic outcomes for a range of conditions, from chemotherapy to hypertension (Weger et al., 2023). Furthermore, developing drugs that target key nodes in this axis (FXR, TGR5, or AhR agonists/antagonists) holds promise for restoring circadian-metabolic homeostasis (Papavasileiou et al., 2023; Gasmi et al., 2025). The challenge here is the complexity of these signaling pathways, where systemic administration could lead to off-target effects, necessitating the development of tissue-specific or time-specific delivery systems. The molecular interplay between the gut microbiome and circadian rhythms is a quintessential example of systems biology. While animal models have been invaluable in revealing potential mechanisms, the path forward requires rigorous human studies to validate these findings, overcome translational hurdles, and develop truly effective, personalized interventions that respect the intricate timing of both host and microbe (Figure 2, Table 1).

**Table 1:** Summary of key research on the molecular interplay between the gut microbiome and circadian rhythms.

**Study objective**	**Study design**	**Main findings**	**Conclusion**	**Reference**
To investigate how host circadian rhythms regulate the gut microbiome.	Review of mechanisms including feeding cycles, immune function, and bile acid secretion.	The host clock drives daily oscillations in microbial composition through rhythmic changes in diet, hormones, and antimicrobial peptide secretion.	The host circadian system exerts top-down control over the gut ecosystem, shaping its structure and function.	(Kubo, 2020; Gutierrez Lopez et al., 2021; Wang et al., 2022; Litwin and Koronowski, 2025; Sharma et al., 2025; Zhu et al., 2025)
To explore how microbial metabolites influence host circadian clocks.	Review of molecular pathways involving SCFAs, bile acids, and tryptophan derivatives.	Microbial metabolites (butyrate, LCA) entrain peripheral clocks via HDAC inhibition, receptor signaling (GPR41/43, FXR), and neuroendocrine pathways.	Gut microbiome-derived metabolites act as circadian zeitgebers, directly influencing host clock gene expression and metabolism.	(Kubo, 2020; Gutierrez Lopez et al., 2021; Alachkar et al., 2022; Khezri et al., 2023; Li et al., 2024; Sharma et al., 2025)
To examine the role of the epithelial clock in colitis.	Used intestinal epithelial-specific *Bmal1* knockout mice challenged with DSS to induce colitis.	Circadian disruption in the gut epithelium led to worse colitis, reduced SCFA-producing bacteria, and increased pro-inflammatory STAT3 signaling.	Gut epithelial circadian dysfunction predisposes to inflammation via microbiome alterations, linking chronodisruption to IBD.	(Jochum et al., 2023)
To determine how a gut microbiome-derived bile acid affects the clock.	In vitro study administering LCA to host intestinal cells.	LCA, a secondary bile acid, lengthened the circadian period by stabilizing the core clock protein CRY2.	A direct molecular pathway exists from a microbial metabolite to the core clock machinery, demonstrating causal microbiome-to-host signaling.	(Pi et al., 2020)
To assess the metabolic effects of time-restricted eating (TRE).	Review of animal and human studies on TRE and intermittent fasting.	TRE restores microbial rhythms, enriches SCFA producers, and improves metabolic health by reinstating a robust circadian metabolic program, independent of caloric intake.	Consolidating eating into a daily window is a powerful intervention to resynchronize the host-microbiome axis and combat metabolic disease.	(Hu et al., 2020; Li et al., 2020)
To understand how circadian dysfunction accelerates cancer metastasis.	Used mouse models of circadian disruption and colorectal cancer (CRC).	Circadian disruption promoted CRC metastasis by increasing myeloid-derived suppressor cells (MDSCs) via gut microbiota-derived taurocholic acid (TCA).	A dysregulated circadian-microbiome axis creates a systemic environment conducive to cancer spread, with TCA as a key mediator.	(Yang and Zhang, 2020)
To explore the role of gut microbes in partitioning metabolism with the liver clock.	Animal studies using genetic and microbiome manipulation techniques.	Gut microbes and the liver clock co-regulate the partitioning of glucose and lipid metabolism across the day-night cycle.	The liver clock and gut microbiome are interdependent partners in maintaining circadian metabolic homeostasis.	(Frazier et al., 2020)
To evaluate the potential of chronopharmacology.	Review of studies on the circadian timing of drug administration.	Drug efficacy and toxicity vary based on the time of day due to circadian rhythms in drug metabolism, microbiome function, and cellular proliferation.	Considering the patient's circadian phase and microbiome rhythms can maximize therapeutic outcomes and minimize side effects.	(Weger et al., 2023)

## 3 Limitations to the gut microbiome and circadian rhythms

The burgeoning field exploring the molecular dialogue between the gut microbiome and circadian rhythms has generated a compelling paradigm for understanding health and disease. However, the complexity of this bidirectional relationship brings forth significant conceptual, methodological, and translational challenges that must be critically acknowledged. These limitations are not merely footnotes but fundamental constraints that shape the interpretation of existing findings and must guide the design of future research. A key weakness in the current literature, which this review also reflects, is a tendency to present mechanistic conclusions from animal studies with a certainty that the overall evidence cannot yet support, without consistently integrating the critical caveats discussed in this section.

### 3.1 Conceptual and mechanistic ambiguities

The most significant conceptual hurdle is the challenge of establishing direct causality over correlation. While numerous studies demonstrate that circadian disruption alters the microbiome and vice versa, untangling the precise sequence of events and the relative contribution of each partner is immensely difficult. The relationship is inherently bidirectional and likely involves positive feedback loops; for instance, circadian disruption causes dysbiosis, which then exacerbates circadian impairment, creating a vicious cycle that is challenging to dissect (Yang et al., 2023a; Moreno-Cortes et al., 2024). Most human evidence remains observational, identifying associations but unable to prove mechanism. While animal models like germ-free or tissue-specific clock gene knockouts are powerful for establishing causality (Mistry et al., 2020; Frazier et al., 2023), they represent extreme, non-physiological states. This limits their ability to accurately reflect the subtler, progressive dysregulations seen in human populations. Furthermore, many interventions, such as TRE, simultaneously affect both systems by altering feeding time, nutrient availability, and microbial ecology, making it nearly impossible to isolate the primary molecular target responsible for the observed benefits (Hu et al., 2020).

### 3.2 Methodological hurdles in measurement and analysis

Substantial methodological challenges plague this field and contribute to inconsistent findings. Accurately measuring circadian parameters in humans is logistically difficult. While core body temperature or dim-light melatonin onset are gold standards, they are invasive and impractical for large-scale studies. Researchers often rely on sleep logs or actigraphy, which are indirect proxies for the central clock and do not capture peripheral circadian rhythms in critical organs like the liver or gut (Weger et al., 2023). Standard 16S rRNA sequencing reveals taxonomic composition but provides limited functional information. While metagenomic sequencing can predict functional potential, it does not confirm the actual production of key metabolites like SCFAs or bile acids. The concentration, bioavailability, and temporal variation of these metabolites are crucial for their biological effect, and measuring these in a time-dependent manner across different host compartments (lumen, portal vein, systemic circulation) is technically demanding and rarely done (Sharma et al., 2025). The immense heterogeneity of the human gut microbiome is a major confounder. An intervention may have profoundly different effects depending on an individual's baseline microbial composition, diet, genetics, and lifestyle, making it difficult to draw universal conclusions and complicating the replication of findings.

### 3.3 The translational gap: from nocturnal rodents to diurnal humans

A critical limitation, often underemphasized in the main narrative of reviews, is the overwhelming reliance on rodent studies for mechanistic insights. Mice are nocturnal animals with profoundly different metabolic rates, dietary habits, gut physiology, and microbiome composition compared to humans. A striking example is the study of bile acids: the bile acid pool and the specific bacterial transformations differ significantly between mice and humans, raising serious questions about the translatability of exciting findings involving microbial bile acid metabolites like LCA or TCA [8, 43]. Furthermore, laboratory mice are typically inbred and housed in controlled, sterile environments, devoid of the complex environmental exposures that shape the human microbiome and circadian system. While necessary for experimental control, this lack of environmental complexity severely limits the generalizability of findings to free-living humans (Litwin and Koronowski, 2025; Xu et al., 2025a).

### 3.4 Temporal and spatial complexity

The system is highly dynamic, yet most studies provide static snapshots. Microbial communities and their metabolomes oscillate throughout the 24-h day (Gutierrez Lopez et al., 2021). A single daily stool sample, the standard in human microbiome studies, provides a poor representation of this diurnal variation and likely misses crucial time-specific interactions (Zhang et al., 2023). The timing of sample collection is therefore a critical, and often unaddressed, confounding variable. The gut microbiome is not homogeneous. Microbial density, composition, and function vary drastically along the gastrointestinal tract and between the lumen and the mucosa. Most human studies analyze fecal samples, which represent the luminal community of the distal colon and may not accurately reflect the mucosal-associated microbiota that interacts more intimately with host immune and epithelial cells (Jochum et al., 2023). Understanding the spatial aspect of these interactions is a major unmet challenge.

### 3.5 Translational hurdles for interventions

Finally, translating these fascinating mechanisms into effective clinical interventions faces significant obstacles. While TRE shows promise, long-term adherence in free-living populations is low. A “one-size-fits-all” approach may not be optimal, as the ideal feeding window likely depends on an individual's chronotype, occupational schedule, and microbial makeup (Godos et al., 2025). Probiotic interventions have yielded notoriously mixed and often disappointing results in humans, failing to replicate robust animal model effects. This is likely because introducing a single bacterial strain into a complex, resilient established community is challenging; the strain may not engraft, or its function may be context-dependent (Li et al., 2020; Du et al., 2025).

**Figure 3:**
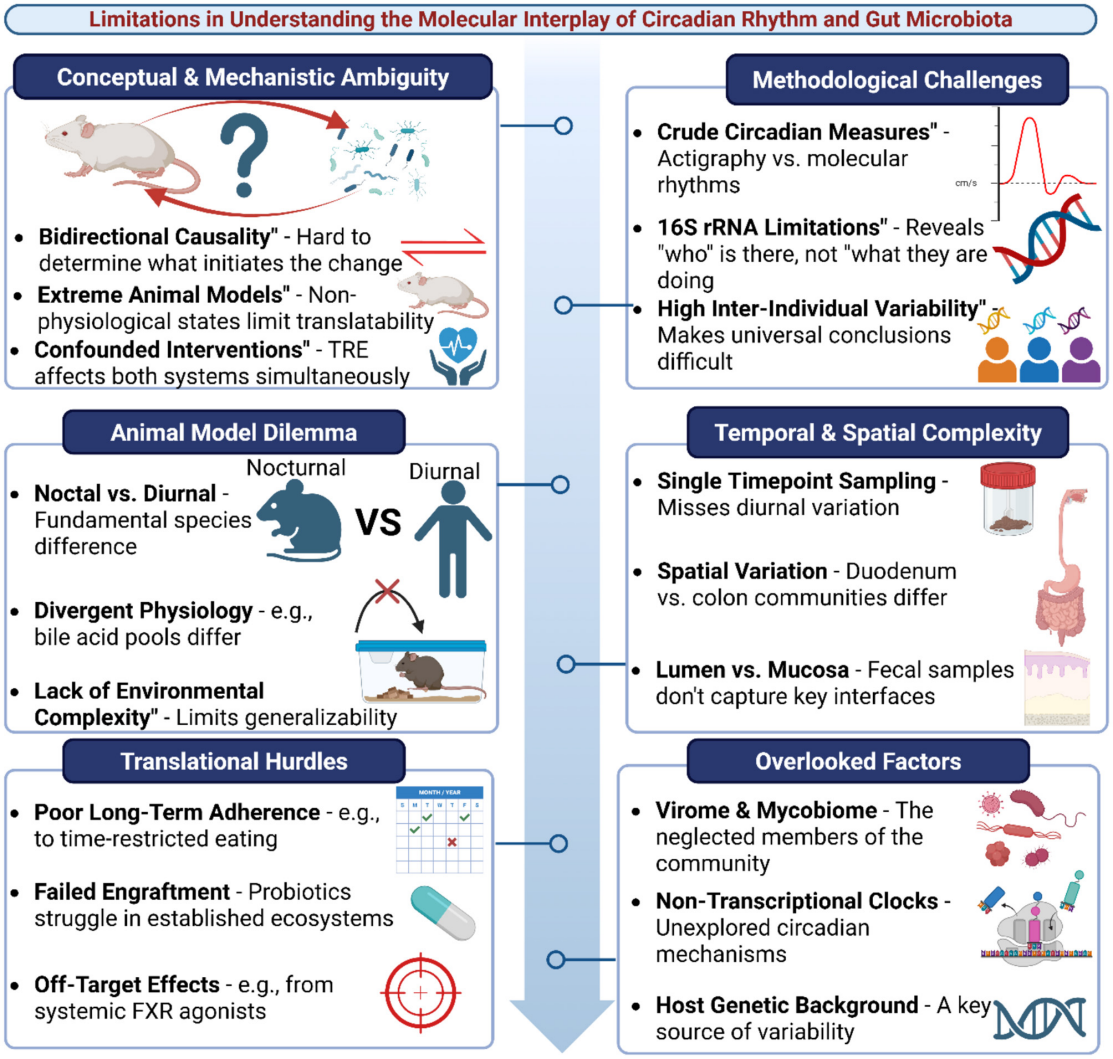
Limitations in understanding the molecular interplay of circadian rhythm and gut microbiota. Multiple conceptual, methodological, and translational challenges hinder progress in elucidating the bidirectional relationship between circadian biology and the gut microbiome. Conceptual and mechanistic ambiguities include bidirectional causality, reliance on extreme animal models, and confounded interventions. Animal model dilemmas arise from nocturnal–diurnal differences, divergent physiology, and reduced environmental complexity, limiting translatability to humans. Methodological challenges include crude circadian measures, limitations of 16S rRNA sequencing, and high inter-individual variability. Temporal and spatial complexities, such as reliance on single timepoint sampling, regional differences along the gut, and lumen vs. mucosa discrepancies, further obscure interpretation. Translational hurdles involve poor long-term adherence, failed probiotic engraftment, and off-target effects of interventions. Additional overlooked factors include the roles of the virome and mycobiome, non-transcriptional circadian clocks, and host genetic background, which all add layers of variability and complexity.

Developing drugs targeting key nodes in this axis (FXR, TGR5 agonists) is promising but fraught with complexity. These receptors are involved in numerous physiological processes, and systemic administration could lead to off-target effects. Achieving tissue-specific or time-specific targeting to mimic natural circadian rhythms is a formidable pharmaceutical challenge (Gumz et al., 2023; Papavasileiou et al., 2023). While the molecular interplay between the gut microbiome and circadian rhythms represents a foundational biological principle, our understanding is still in its infancy. It is constrained by conceptual complexities, methodological inadequacies, and significant translational barriers. Acknowledging these limitations is not to dismiss the compelling hypothesis but to define the frontiers of current knowledge and underscore the need for a more critical and integrated interpretation of the evidence. Future research must prioritize longitudinal human studies with dense temporal and spatial sampling, develop more sophisticated tools for in vivo measurement, and create integrative models that account for the full complexity of the host-microbe-clock axis (Figure 3, Table 2).

**Table 2:** Summary of microbial species and metabolites in disease pathogenesis and intervention.

**Disease category**	**Associated microbial species**	**Key metabolites involved**	**Effect**	**Mechanism**	**Reference**
Metabolic disease (Obesity, T2DM)	• Reduced SCFA-producers (*Roseburia, Faecalibacterium*) • Increased *Firmicutes*/*Bacteroidetes* ratio (dysbiosis)	• Decreased SCFAs (Butyrate, Propionate, Acetate) • Altered Bile Acid pool (reduced FXR agonists)	Occurrence	Dysbiosis and loss of rhythmic SCFA production impair insulin sensitivity, glucose tolerance, and circadian metabolic coordination.	(Hu et al., 2020; Frazier et al., 2023; Yuan et al., 2025b)
	• Increased SCFA-producers (*Akkermansia muciniphila, Roseburia*)	• Increased SCFAs (Butyrate, Propionate)	Improvement	TRE enriches SCFA-producers. SCFAs improve metabolic health via GPR41/43 signaling, HDAC inhibition, and reinforcing circadian rhythms.	(Hu et al., 2020; Li et al., 2020; Gutierrez Lopez et al., 2021)
Gastrointestinal disorders (IBD)	• Reduced SCFA-producers (*Faecalibacterium prausnitzii*) • General dysbiosis	• Decreased SCFAs (especially Butyrate)	Occurrence	Circadian disruption in gut epithelium reduces SCFA levels, leading to impaired barrier function, STAT3-driven inflammation, and worsened colitis.	(Jochum et al., 2023)
	• Increased SCFA-producers	• Increased SCFAs (Butyrate)	Improvement	Butyrate serves as the primary energy source for colonocytes, strengthens the gut barrier, and has anti-inflammatory effects, helping to resolve inflammation.	(Jochum et al., 2023; Sharma et al., 2025)
Neurodegenerative disorders (AD, PD)	• General dysbiosis & reduced microbial diversity • Pro-inflammatory species	• Increased LPS (pro-inflammatory) • Altered Tryptophan metabolites (Serotonin/Indoles)	Occurrence	Dysbiosis promotes neuroinflammation, impairs glymphatic clearance (during sleep), and may contribute to Aβ/tau pathology in AD and α-synuclein aggregation in PD.	(Gumz et al., 2023; Khezri et al., 2023; Moreno-Cortes et al., 2024)
	• Beneficial species (*Lactobacillus, Bifidobacterium* strains)	• Increased SCFAs • Balanced neuroactive metabolites (Serotonin)	Improvement	Specific probiotics and TRE can modulate neuroinflammation, improve sleep, and support the production of metabolites that positively influence the gut-brain axis.	(Alachkar et al., 2022; Du et al., 2025)
Cancer (colorectal cancer)	• Dysbiotic communities • Specific pathogens	• Increased Taurocholic acid (TCA)	Occurrence	Circadian disruption promotes a microbiome that produces TCA, which drives the accumulation of immunosuppressive MDSCs, facilitating cancer metastasis.	(Yang and Zhang, 2020)
	• Restoration of a balanced microbiota (via FMT)	• Normalization of oncogenic metabolite levels (reduced TCA)	Improvement	Interventions that restore a healthy, rhythmic microbiome can reduce the production of pro-tumorigenic metabolites and re-establish anti-tumor immunity.	(Yang and Zhang, 2020; He et al., 2024)

## 4 Prospective studies for the gut-microbiome-circadian axis

The compelling, yet complex, paradigm of a bidirectional gut-microbiome-circadian dialogue presents a fertile ground for future research. While significant progress has been made in identifying associations, the field must now pivot from correlation to causation and from mechanistic insight to therapeutic application. The following prospective research agenda is structured to overcome the specific limitations outlined in the previous section, moving beyond a disjointed list to present a coordinated, interdisciplinary strategy. This framework prioritizes studies that establish causality, resolve spatiotemporal dynamics, enable personalization, and expand the scope of inquiry, with each proposal explicitly linked to the experimental designs required to fill critical knowledge gaps.

### 4.1 Priority area 1: establishing definitive causality and mechanism

A primary and immediate goal is to dismantle the correlative nature of much current evidence. This requires studies that can delineate the precise sequence of molecular events and identify the essential actors.

#### 4.1.1 Study 1.1: longitudinal multi-omics in a controlled human chronodisruption model

To move beyond observational human studies and animal models, this research proposes a controlled human laboratory intervention where healthy participants undergo a simulated shift work or chronic jet lag protocol, enabling the tracking of the temporal sequence of events during circadian disruption. Throughout baseline, disruption, and recovery phases, high-frequency blood, stool, and saliva samples will be analyzed using a multi-omics pipeline, including transcriptomics of PBMCs, metagenomics of the microbiome, metabolomics, and proteomics to assess systemic rhythms, microbial shifts, and host inflammatory and metabolic responses. We hypothesize that circadian misalignment will induce rapid, reproducible alterations in the microbial metatranscriptome and metabolome prior to major taxonomic shifts, and that these metabolic changes will directly correlate with and predict the dampening of host metabolic and immune pathway rhythms, resulting in a detailed temporal map that establishes a causal sequence from clock disruption to microbial functional change to host physiological consequence.

#### 4.1.2 Study 1.2: targeted metabolite rescue in circadian-disrupted hosts.

To provide direct proof that specific microbial metabolites are causal zeitgebers, this research proposes a targeted approach to test their administration in animal models or human organoids. Defined compounds, such as the secondary bile acid LCA or specific SCFA cocktails, would be administered at a consistent time of day, coupled with genetic knockout models to identify essential host receptors like FXR, TGR5, or AhR. We hypothesize that this timed metabolite administration will rescue specific aspects of circadian disruption such as restoring liver clock amplitude or improving glucose tolerance, in a receptor-dependent manner, with readouts including high-resolution circadian gene expression and metabolic and immune markers. The expected outcome is conclusive evidence for (or against) defined microbial metabolites as causal mediators, thereby pinpointing precise molecular targets for future drug development.

### 4.2 Priority area 2: high-resolution spatiotemporal mapping

Understanding where and when interactions occur is critical, as fecal samples and single time points provide an incomplete picture.

#### 4.2.1 Study 2.1: a diurnal atlas of the gut-liver-brain metabolome

To address the critical limitation that fecal samples inadequately represent the location-dependent bioavailability of metabolites at their sites of action, this study proposes a spatiotemporal investigation in animal models involving precisely timed sampling every 4–6 h over 24 h from luminal contents, mucosal scrapings, portal and systemic blood, and liver tissue under both normal and circadian-disrupted conditions, with advanced mass spectrometry used to create quantitative metabolite profiles across these compartments. We hypothesize that microbial metabolite rhythmicity will be most pronounced at the intestinal mucosal interface and within the portal vein, and that this rhythm will be significantly blunted during circadian disruption, leading to dysfunctional signaling to the liver; the expected outcome is a high-resolution spatiotemporal atlas identifying which metabolites are delivered rhythmically to target organs and how this crucial dynamic is compromised in disease.

#### 4.2.2 Study 2.2: single-cell circadian transcriptomics of the gut epithelium

To resolve how the diverse cell types of the gut epithelium the primary host-microbiome interface, individually respond to microbial timing cues, this study would employ single-cell RNA sequencing on intestinal epithelial cells isolated from mice at multiple times across a 24-hour cycle, comparing germ-free, conventionally raised, and metabolite-treated models. We hypothesize that distinct epithelial cell types, such as enteroendocrine, goblet, and Paneth cells, will exhibit unique circadian transcriptomes and differential responsiveness to microbial signals, an outcome that will identify the most responsive cellular sensors and refine our mechanistic understanding of host-microbe cross-talk.

### 4.3 Priority area 3: translation and personalization

Bridging the gap to clinical application requires a focus on individual variability and the development of sophisticated interventions.

#### 4.3.1 Study 3.1: chrono-microbiome phenotyping for personalized nutrition

Recognizing that high inter-individual variability in microbiome composition is a likely key determinant of response to TRE, this research proposes a large-scale human clinical trial where participants undergo deep phenotyping, including microbiome sequencing, metabolomics, genotyping, and actigraphy before being randomized to different TRE windows. We hypothesize that baseline microbial features, such as the abundance of SCFA-producers or bile acid metabolism gene capacity, will predict an individual's optimal TRE window and their degree of metabolic improvement, with the outcome being a validated predictive model for personalizing chrono-nutrition based on an individual's unique “chrono-microbiome” profile.

#### 4.3.2 Study 3.2: Engineering next-generation chronobiotics

To overcome the limitations of conventional probiotics, namely their poor engraftment and lack of circadian functionality, this project proposes using synthetic biology to engineer model commensal bacteria with genes for beneficial metabolites, such as butyrate, placed under the control of a promoter inducible by a host feeding-time signal. These engineered “chronobiotics” would be tested in circadian-disrupted animal models, with the hypothesis that a time-controlled, phased release of metabolites will enhance host rhythm entrainment and metabolic health more effectively than constitutive production, thereby providing proof-of-concept for a novel therapeutic class of microbes designed specifically to reinforce the host circadian system.

### 4.4 Priority area 4: expanding the scope of inquiry

The field must look beyond bacteria and core metabolism to fully understand the system.

#### 4.4.1 Study 4.1: the microbiome-circadian axis in neurodegenerative pathogenesis

To address the mechanistically vague link between sleep loss, neurodegeneration, and the microbiome, this study would subject animal models of Alzheimer's or Parkinson's disease to chronic circadian disruption, with or without concomitant antibiotic treatment or FMT from healthy donors, while tracking pathology, neuroinflammation, cognitive function, and multi-omics profiles. We hypothesize that circadian disruption accelerates pathology and cognitive decline in a microbiome-dependent manner, mediated by impaired glymphatic clearance and microbial metabolite-driven neuroinflammation, an outcome that would provide a mechanistic explanation and position the gut microbiome as a critical therapeutic target for preventing neurodegeneration.

#### 4.4.2 Study 4.2: the role of the virome and mycobiome in circadian regulation

To address the significant gap in circadian microbiome research which has almost exclusively focused on bacteria, neglecting other kingdoms, this study proposes longitudinal metagenomic and ITS sequencing on samples from human chronodisruption studies and animal models to characterize the virome and mycobiome, combined with targeted viral depletion or antifungal treatments to perturb these communities and observe the effects on host circadian rhythms. We hypothesize that the virome and mycobiome exhibit their own diurnal rhythms and play a previously overlooked role in stabilizing bacterial communities and influencing host circadian immunity, with the outcome being the first comprehensive map of multi-kingdom circadian dynamics, potentially revealing new regulatory entities for the circadian system.

### 4.5 An integrated path forward

**Figure 4:**
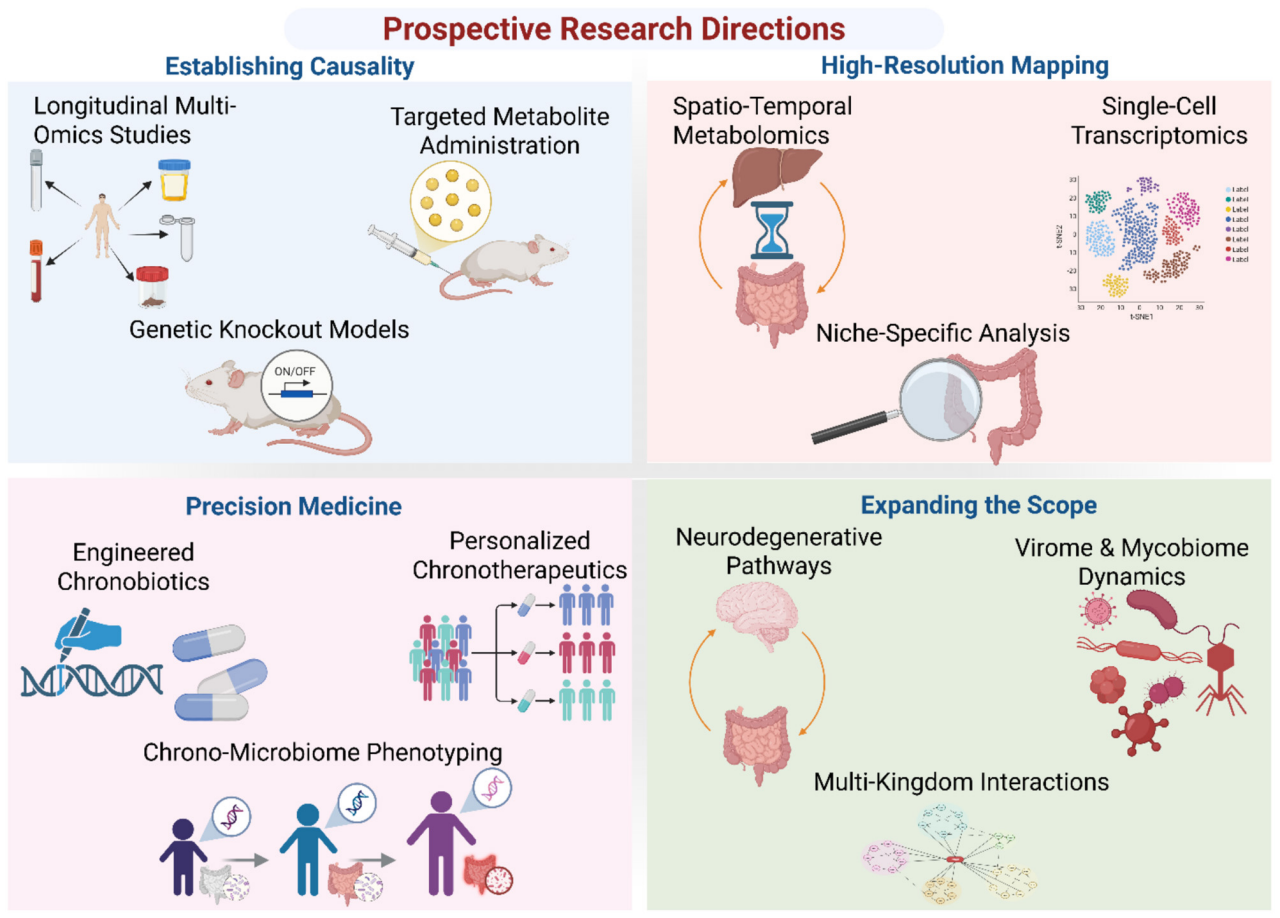
Key thematic areas for future research on the gut microbiome-circadian rhythm axis. This conceptual figure outlines four prospective research directions designed to move from correlation to causation and toward therapeutic translation. Panel 1: Establishing Causality focuses on mechanistic studies using longitudinal multi-omics in humans, targeted metabolite administration, and genetic knockout models to define hierarchical relationships. Panel 2: High-Resolution Mapping highlights the need for spatio-temporal metabolomics across host compartments, single-cell transcriptomics of the gut epithelium, and niche-specific analysis to move beyond fecal sampling. Panel 3: Precision Medicine envisions the translation of this knowledge through chrono-microbiome phenotyping for personalized nutrition, the development of engineered chronobiotics, and tailored chronotherapeutics. Panel 4: Expanding the Scope looks beyond bacteria to explore the role of the axis in neurodegenerative pathways and to characterize the circadian dynamics of the virome and mycobiome within a multi-kingdom framework.

The proposed studies represent a necessary evolution for the field, forming a cohesive and prioritized research agenda rather than a simple list. By strategically focusing on causality (Area 1), resolution (Area 2), translation (Area 3), and expansion (Area 4), future research can systematically decode the molecular language of the microbiome-clock dialogue. Success in these endeavors will require collaboration across chronobiology, microbiology, bioinformatics, and clinical medicine, but the payoff will be a precise and actionable understanding that unlocks the full potential of chrono-microbiome medicine (Figure 4, Table 3).

**Table 3:** Proposed prospective research agenda for the gut-microbiome-circadian axis.

**Study title**	**Hypothesis**	**Objective**	**Study population/model**	**Materials and methods**	**Predicted findings**
Longitudinal multi-omics in a controlled human chronodisruption model	Circadian misalignment induces rapid alterations in the microbial metatranscriptome and metabolome prior to taxonomic shifts, and these changes predict the dampening of host metabolic and immune rhythms.	To establish a temporal causal sequence from clock disruption to microbial functional change to host physiological consequence.	Healthy human participants in a controlled laboratory setting.	Simulated shift work/jet lag protocol. High-frequency sampling (blood, stool, saliva). Multi-omics analysis: transcriptomics (PBMCs), metagenomics, metabolomics, proteomics.	A detailed map showing microbial metabolic dysfunction precedes and drives host rhythm disruption, identifying key early biomarker pathways.
Targeted metabolite rescue in circadian-disrupted hosts	Timed administration of specific microbial metabolites (LCA, SCFAs) will rescue aspects of circadian disruption in a receptor-dependent manner.	To provide direct proof that specific microbial metabolites are causal circadian zeitgebers and identify their essential host receptors.	Animal models (circadian-disrupted mice) or human organoids; genetic knockout models (FXR, TGR5, AhR).	Timed administration of defined metabolites (LCA, SCFA cocktails). Readouts: high-resolution circadian gene expression, metabolic profiles (glucose tolerance), immune markers.	Conclusive evidence for specific metabolites as causal mediators. Restoration of liver clock amplitude and metabolic function only in the presence of essential receptors.
A diurnal atlas of the gut-liver-brain metabolome	Microbial metabolite rhythmicity is most pronounced at the intestinal mucosa and portal vein, and is blunted during circadian disruption, leading to dysfunctional signaling to the liver.	To create a high-resolution spatiotemporal atlas of microbial metabolite flux to target organs under normal and disrupted conditions.	Animal models (mice or rats).	Precisely timed sampling (every 4-6 hrs) over 24h from lumen, mucosa, portal blood, systemic blood, liver. Advanced mass spectrometry for quantitative metabolomics.	Identification of which key metabolites are delivered rhythmically to the liver; revelation of how this dynamic communication is compromised in chronodisruption.
Single-cell circadian transcriptomics of the gut epithelium	Distinct intestinal epithelial cell types exhibit unique circadian transcriptomes and differential responsiveness to microbial signals.	To identify the most responsive cellular sensors in the gut epithelium and refine the mechanistic understanding of host-microbe cross-talk.	Intestinal epithelial cells from mouse models: Germ-free, conventionally raised, Metabolite-treated.	Single-cell RNA sequencing (scRNA-seq) on cells isolated at multiple time points across a 24-hour cycle.	A cell-type-specific map of circadian and microbial responsiveness, identifying key sensor cells (enteroendocrine, goblet) and their regulated pathways.
Chrono-microbiome phenotyping for personalized nutrition	Baseline microbial features (SCFA-producer abundance, bile acid metabolism genes) predict an individual's optimal TRE window and metabolic improvement.	To develop a validated predictive model for personalizing TRE based on an individual's “chrono-microbiome” profile.	Human participants in a large-scale clinical trial.	Deep phenotyping (microbiome sequencing, metabolomics, genotyping, actigraphy). Randomization to different TRE windows.	A algorithm that matches an individual's microbiome profile to an ideal feeding window, maximizing metabolic outcomes like weight loss and insulin sensitivity.
Engineering next-generation chronobiotics	A time-controlled, phased release of metabolites from engineered bacteria will enhance host rhythm entrainment and metabolic health more effectively than constitutive production.	To provide proof-of-concept for a novel therapeutic class of microbes designed specifically to reinforce the host circadian system.	Circadian-disrupted animal models (mice).	Synthetic biology to engineer commensal bacteria with metabolite genes under a host feeding-time-inducible promoter. Testing in animal models.	Engineered “chronobiotics” successfully engraft and show superior efficacy in restoring circadian rhythms and metabolic health compared to conventional probiotics.
The microbiome-circadian axis in neurodegenerative pathogenesis	Circadian disruption accelerates neurodegeneration in a microbiome-dependent manner, mediated by impaired glymphatic clearance and metabolite-driven neuroinflammation.	To provide a mechanistic explanation linking sleep loss, the microbiome, and neurodegeneration, positioning the gut as a therapeutic target.	Animal models of Alzheimer's or Parkinson's disease.	Chronic circadian disruption with/without antibiotic treatment or FMT from healthy donors. Readouts: pathology, neuroinflammation, cognitive tests, multi-omics.	Circadian disruption worsens pathology only in the presence of a microbiome, identifying specific microbial pathways and metabolites that drive neuroinflammation and cognitive decline.
The role of the virome and mycobiome in circadian regulation	The gut virome and mycobiome exhibit diurnal rhythms and play a previously overlooked role in stabilizing bacterial communities and influencing host circadian immunity.	To create the first comprehensive map of multi-kingdom circadian dynamics and reveal new regulatory entities for the circadian system.	Human participants from chronodisruption studies; Animal models (mice).	Longitudinal metagenomic & ITS sequencing. Targeted viral depletion or antifungal treatments to perturb non-bacterial kingdoms.	Discovery of rhythmic viral and fungal populations that correlate with, and functionally impact, host circadian gene expression and immune function.

## 5 Conclusion

The evidence synthesized in this review unequivocally establishes that the molecular interplay between the gut microbiome and circadian rhythms is a fundamental pillar of human physiology, moving beyond mere association to reveal a framework of reciprocal causation. This dialogue, mediated by a sophisticated exchange of microbial metabolites SCFAs, bile acids, and tryptophan derivatives ensures the precise temporal coordination of metabolism, immunity, and neurological function. However, as the limitations and prospective studies sections highlight, our understanding is still maturing. The overreliance on rodent models, the challenge of establishing direct causality in humans, and the conspicuous gap in foundational literature from the pioneers of this field remind us that this is a dynamic, not a settled, area of science.

The disruption of this axis, driven by modern lifestyle factors such as artificial light at night, erratic eating, and shift work, serves as a critical pathway to a spectrum of chronic diseases. Yet, it is the very nature of this bidirectional relationship where dysbiosis and chronodisruption form a vicious cycle that reveals its therapeutic potential. Breaking this cycle requires interventions that simultaneously engage both systems. In this regard, TRE emerges as a powerful, accessible non-pharmacological strategy to resynchronize host-microbe rhythms and improve metabolic health. However, its long-term efficacy will depend on overcoming adherence challenges and moving toward personalized feeding windows.

Looking forward, the promise of this field lies not in generic solutions but in precision medicine. The future belongs to leveraging individual “chrono-microbiome” profiles to tailor nutritional strategies, probiotic interventions, and drug timing (chronopharmacology) for maximal benefit. This review has critically appraised the current evidence, noting where exciting findings, such as the direct stabilization of CRY by LCA, are still preliminary and require validation. The path ahead must prioritize human studies that translate compelling mechanistic insights from animal models into effective, personalized clinical applications. This necessitates a research agenda focused on establishing causality, resolving spatiotemporal dynamics, and developing next-generation therapeutics like engineered chronobiotics.

Ultimately, this review underscores that our health is intrinsically linked to the rhythmic harmony we maintain with our microbial partners. Acknowledging this partnership is the first step toward a new era of medicine that treats time not as a constant, but as a central, modifiable determinant of wellbeing. The goal is clear: to harness the intricate language of the gut-clock axis to develop strategies that restore rhythmic harmony, thereby combating the rising tide of chronic disease rooted in the disarray of our internal clocks and the ecosystems within us.
